# Unraveling attachment – A network analysis of the cognitive pathways linking attachment and prolonged grief

**DOI:** 10.1017/S0033291725101669

**Published:** 2025-09-19

**Authors:** Kirsten V. Smith, Nora Skjerdingstad, Omid V. Ebrahimi, Fiona Maccallum, Anke Ehlers

**Affiliations:** 1 Department of Experimental Psychology, University of Oxford, Oxford, UK; 2 Department of Psychiatry, University of Oxford, Oxford, UK; 3 School of Psychology, University of Queensland, Brisbane, Australia; 4 Oxford Health NHS Foundation Trust, Oxford, UK.

**Keywords:** appraisals, attachment, bereavement, cognitive-behavioural mechanisms, coping strategies, grief, memory characteristics, network analysis, prolonged grief disorder, social disconnection

## Abstract

**Background:**

Attachment style is widely recognized as influential in shaping responses to bereavement and prolonged grief disorder (PGD). Although theorized extensively, empirical clarity regarding how attachment styles specifically impact PGD symptoms and therapeutic implications remains limited. This study aimed to identify cognitive-behavioral mechanisms linking attachment styles to PGD symptoms.

**Methods:**

Data were collected from a community sample of 695 bereaved adults. Network analysis explored interactions between attachment styles (anxious and avoidant) and various cognitive-behavioral factors associated with PGD, including appraisals, memory characteristics, maladaptive coping strategies, and a sense of social disconnection.

**Results:**

The findings reveal attachment styles as peripheral within the network, suggesting that their direct influence on PGD symptoms may be less central than previously theorized. However, anxious attachment correlated positively with injustice rumination and altered social self, while avoidant attachment was positively associated with perceived loss of future and relationships and preferences for solitude, and negatively associated with proximity-seeking behaviors and fear of losing connection to the deceased. Cognitive-behavioral factors, particularly memory characteristics and social disconnection, held central positions within the network, mediating relationships between attachment styles and PGD.

**Conclusions:**

Attachment styles indirectly influence PGD through cognitive-behavioral pathways rather than exerting strong direct effects. By bridging the gap between attachment theory and cognitive-behavioral approaches to grief, this study offers a more nuanced understanding of its relationship with PGD and points toward potential new avenues for future interventions aimed at addressing attachment-related challenges in bereaved individuals.

## Introduction

Prolonged grief disorder (PGD) is characterized by intense, persistent grief symptoms exceeding culturally accepted mourning periods, and significantly impairing daily functioning (Prigerson, Boelen, Xu, Smith, & Maciejewski, 2021). PGD affects approximately 5–16% of bereaved individuals, particularly after traumatic or sudden losses (Comtesse et al., 2024; Djelantik, Smid, Mroz, Kleber, & Boelen, 2020). It substantially impacts mental health, frequently co-occurring with severe issues like increased suicide risk (Komischke-Konnerup, Zachariae, Johannsen, Nielsen, & O’Connor, 2021; Smith & Ehlers, 2021b), reduced social functioning (Smith, Wild, & Ehlers, 2020b), and overall quality of life (Rodriguez-Villar et al., 2024), highlighting its importance for research and clinical interventions.

Attachment theory, specifically attachment styles, has long been thought to shape the nature of grief responses (Houwen et al., 2010; Shear & Shair, 2005; Stroebe, Schut, & Stroebe, 2005) and has been influential to a number of theoretical models of PGD constructs (Maccallum & Bryant, 2013; Shear et al., 2007). Broadly, attachment can be conceptualized along two dimensions: anxious and avoidant. Individuals with an anxious attachment style tend to fear abandonment and often seek reassurance and closeness, whereas those with an avoidant style are inclined to favor interpersonal distance over closeness in relationships (Fraley & Shaver, 2000). Attachment anxiety is thought to contribute to poor bereavement outcomes by fostering cognitive hypervigilance and proximity seeking toward the deceased, reinforcing beliefs that one is less able to cope with one’s own, undermining self-regulation and integration of the loss memory. Attachment avoidance, on the other hand, which is thought to foster disengagement and diversion of attention away from emotionally threatening information, may be adaptive in the short term but has also been proposed to place one at risk of poor mental health outcomes after a bereavement over the longer term by limiting access to social supports that may help with adjustment to the loss (Fraley, Davis, & Shaver, 1998; Fraley & Shaver, 1997; Mikulincer & Shaver, 2008, 2022).

Systematic reviews (Eisma, Bernemann, Aehlig, Janshen, & Doering, 2023; Russ, Stopa, Sivyer, Hazeldine, & Maguire, 2024) support the association between attachment anxiety and higher concurrent PGD symptoms. However, associations with attachment avoidance have been inconsistent, with some studies reporting positive relationships and others not. Moreover, as most studies have only considered bivariate relationships between attachment and PGD, there remains much to be learned about ‘how’ these attachment processes influence PGD severity. For example, Smith and Ehlers (2020) found baseline attachment styles predicted PGD symptoms at 6 and 12 months postbereavement, but this association vanished after controlling for cognitive behavioral variables. These findings raise the possibility that rather than having a direct impact on PGD symptoms, attachment style may indirectly influence PGD by shaping cognitive and behavioral processes surrounding loss.

Boelen and Klugkist (2011) reported mediation effects by factors such as a sense of unrealness about the loss, avoidance behaviors, and negative beliefs, while Kho, Kane, Priddis, and Hudson (2015) found anxious attachment particularly linked to difficulty accepting loss. Taken together, these findings suggest that while anxious and avoidant attachment styles are often associated with more intense or persistent grief, the strength and specificity of these relationships depend on mediating cognitive, behavioral factors. Clarifying these indirect pathways is crucial for developing targeted, evidence-based interventions addressing attachment dynamics in bereavement.

Unraveling these relationships requires advanced analytic techniques. Traditional regression analyses focus primarily on direct links between predictors and outcomes, potentially missing complex interrelations within psychological phenomena like grief. Network analysis, however, provides a robust method for examining simultaneous direct and indirect relationships, revealing concurrent interactions among underlying mechanisms (Borsboom & Cramer, 2013; McNally et al., 2015). Early network studies highlighted mental health disorders as emergent processes rather than latent constructs, demonstrating symptom amplification and comorbidity (Cramer, Waldorp, Van Der Maas, & Borsboom, 2010; Maccallum, Malgaroli, & Bonanno, 2017; Malgaroli, Maccallum, & Bonanno, 2018). For example, Robinaugh, LeBlanc, Vuletich, and McNally (2014)) showed PGD symptoms that were more interrelated with each other than with depression symptoms, validating PGD as distinct. Other studies identified candidate variables for comorbidity, for example, emotional pain was highlighted as the most central symptom of PGD, and along with loneliness and meaninglessness bridged the gap between grief and depression (Maccallum et al., 2017; Malgaroli et al., 2018). Network analyses have expanded to incorporate broader psychopathology features; for example, Maccallum and Bryant (2020) identified PGD symptoms most impacting quality of life. We extend this approach to explore network associations between cognitive behavioral mechanisms, attachment styles, and PGD. A key implication of this approach is that identifying central nodes can highlight the most effective targets to disrupt network dynamics and alleviate symptoms, providing insights for targeted psychological interventions (Borsboom & Cramer, 2013; Epskamp, Borsboom, & Fried, 2018; Malgaroli, Maccallum, & Bonanno, 2022; McNally et al., 2015; Robinaugh, Millner, & McNally, 2016).

The *Oxford Grief Study*, involving over 1,000 bereaved participants across two datasets, has provided valuable insights into cognitive-behavioral mechanisms influencing PGD development and maintenance. This research identified key roles for grief-related appraisals, a sense of social disconnection, maladaptive coping strategies, and memory characteristics (Smith & Ehlers, 2020). For example, loss-related memory characteristics were strongly associated with later PGD symptoms, even after controlling for prior symptoms and autocorrelations (Smith, Wild, & Ehlers, 2022). These characteristics typify loss memories that are intrusive, vivid, have a ‘sense of nowness’ and significant visceral consequences, are easily triggered, predominantly negative in nature, and evoke negative emotions, even when the memories themselves may be objectively positive. The scale measuring these features was developed based on interviews with bereaved people with and without PGD and assessed how well a loss memory has been integrated into one’s broader autobiographical memory (Smith, Rankin, & Ehlers, 2020a). Maladaptive coping strategies – including avoidance, proximity seeking, loss rumination, and injustice rumination – also predicted later PGD (Smith, Wild, & Ehlers, 2024). Additionally, negative grief-related appraisals about the self, life, future, relationships, catastrophic consequences, regret, and maintaining a connection with the deceased predicted severe, enduring grief trajectories (Smith & Ehlers, 2020). A sense of social disconnection consistently predicted PGD across studies, encompassing feelings of social alteration, negative beliefs about others’ responses to grief, and a perceived safety in solitude (Rodriguez-Villar et al., 2024; Smith, Wild, & Ehlers, 2020b; Wanza et al., 2023). Despite an extensive study of these cognitive-behavioral factors in relation to PGD, empirical links to attachment styles remain largely theoretical.

Building on this work, the current study aimed to explore associations among cognitive-behavioral mechanisms identified in the Oxford Grief Study, attachment styles (anxious and avoidant), and PGD symptoms. Based on prior findings (Smith et al., 2022, 2024; Smith & Ehlers, 2020; Smith & Ehlers, 2021a; Smith & Ehlers, 2021b; Smith, Wild, & Ehlers, 2020b), we hypothesized direct associations between PGD symptoms and cognitive-behavioral processes (appraisals, memory characteristics, coping strategies), with attachment styles exerting indirect effects through these mechanisms. By identifying which factors are most strongly interrelated, our approach aims to clarify the underlying pathways that contribute to the maintenance of prolonged grief and to highlight promising targets for psychological intervention.

## Methods

### Participants

A total of 695 adults who had been bereaved for a minimum of 6 months prior to participating completed the questionnaires (M = 56.79 months, SD = 80.86, range = 6–685 months). Recruitment was conducted through bereavement charity mailing lists, targeted social media advertisements, and the Google content network. No upper limit was placed on time since loss. To be eligible, respondents had to name the deceased as a close loved one, excluding acquaintances or distant relatives.

#### Symptom measures

*The Prolonged Grief Disorder Inventory* (PG-13; Prigerson & Maciejewski, 2008) assesses separation distress together with related emotional, cognitive, and behavioral difficulties, capturing both intensity and duration. Each item is rated 1 = not at all to 5 = nearly every day for the previous month. We used an expanded version covering the 10 DSM-5-TR symptoms (for further details, see Smith et al., 2022). A probable PGD diagnosis required ≥1 separation-distress symptom, ≥3 of eight daily-disturbance symptoms, and marked social, occupational or domestic impairment (Prigerson et al., 2009). To satisfy ICD-11 criteria, PCL-5 item 14 (difficulty experiencing positive emotions) was rescaled and added (Killikelly & Maercker, 2018). Internal consistency was excellent across definitions (ICD α = 0.90; DSM α = 0.92).

#### Attachment

Attachment anxiety and avoidance were measured with the 12-item *Experiences in Close Relationships Scale – Revised* (Wei, Russell, Mallinckrodt, & Vogel, 2007). Items are rated 1 = strongly disagree to 7 = strongly agree. Six items index anxiety (e.g. ‘I need a lot of reassurance that I am loved by my close loved ones’) and six index avoidance (e.g. ‘I do not often worry about being abandoned’). Both subscales showed adequate reliability in this sample (anxious α = 0.79; avoidant α = 0.79).

### Cognitive measures: The Oxford Grief Measures

#### Loss-related memory characteristics scale (OG-M)

This 27-item measure uses a 5-point scale (0 = *not at all* to 4 = *very strongly*) (Smith et al., 2022). Twenty-three items address memory triggers and their impacts (e.g. ‘I am reminded of the loss for no apparent reason’), the nature of memories (e.g. ‘Memories of things we did together are painful’), difficulties recalling positive experiences (e.g. ‘I struggle to remember positive times without [−]’), and visceral consequences of these memories (e.g. ‘Memories of [−]‘s death make my body ache with overwhelming fatigue’). Four additional items assess involuntary memories of the loss, specifically their distressing nature and the sense of occurring in the present rather than the past. The OG-M demonstrated excellent reliability (α = 0.95).

#### Negative grief appraisals (OG-A)

This 35-item questionnaire measures negative cognitive appraisals of grief across five domains: loss of self and life (e.g. ‘I have lost my sense of who I am in the world’; α = 0.93), regret (e.g. ‘I blame myself for things I did or did not do when [−] was alive’; α = 0.82), catastrophic grief consequences (e.g. ‘If I start to cry, I won’t be able to stop’; α = 0.86), loss of relationships and future (e.g. ‘I cannot maintain previous relationships without [−]’; α = 0.89), and fear of losing connection to the deceased (e.g. ‘If I don’t do everything I can to feel close to [−], I will lose them forever’; α = 0.89) (Smith, 2018). Items are rated on a 7-point Likert scale (1 = *totally disagree*, 7 = *totally agree*). The measure demonstrated excellent overall reliability (α = 0.96).

#### Coping strategies (OG-CS)

The OG-CS is a 23-item measure assessing the frequency of grief-related coping strategies used in the past month, rated on a 5-point scale (1 = *never*, 5 = *always*) (Smith et al., 2024). It covers four domains: avoidance (α = 0.73), proximity seeking (α = 0.84), loss rumination (α = 0.89), and injustice rumination (α = 0.83). The scale demonstrated excellent overall reliability (α = 0.92).

#### Social disconnection (OG-SD)

This 15-item scale assesses bereaved individuals’ appraisals of social disconnection over the past month, rated on a 7-point Likert scale (1 = *totally disagree*, 7 = *totally agree*) (Smith, Wild, & Ehlers, 2020b). It covers three domains: negative interpretations of others’ reactions (3 items, e.g. ‘If I show my real feelings other people will think I am not normal’; α = 0.79), altered social self (8 items, e.g. ‘I can’t be myself around other people the way I used to’; α = 0.92), and safety in solitude (4 items, e.g. ‘It is easier to be alone than to have to pretend to feel ok’; α = 0.89). The overall internal consistency was excellent (α = 0.94).

### Data analysis

#### Statistical analysis

Statistical analyses were conducted in R (version 4.4.1; R core team, 2024) with code available through the Open Science Farmwork (OSF): https://osf.io/h25y3/?view_only=aedd5ec2b43044e08a5f65a3e351de19. Multivariate Gaussian Graphical Models (GGMs; Epskamp, Borsboom, & Fried, 2018) were estimated to visualize networks of partial correlations using the bootnet package (Epskamp, Borsboom, & Fried, 2018). Nodes represent individual variables, connected by edges whose widths indicate partial correlation strengths after controlling for other variables. Node values were derived from each scale’s empirically supported factor analyses – this resulted in total sum scores for the PGD and memory characteristics measures and subscale sum scores for the other questionnaires.

Analysis proceeded in steps:

1. The *goldbricker* function from the *networktools* R-package (Jones & Jones, 2018) assessed empirical overlap among theoretically selected variables; no redundant variables were identified.
2. Networks were estimated using estimateNetwork from bootnet (Epskamp, Borsboom, & Fried, 2018). The graphical ‘least absolute shrinkage and selection operator’ (LASSO) regularization algorithm (glasso; Friedman, Hastie, & Tibshirani, 2008) combined with Extended Bayesian Information Criterion (EBIC; Chen & Chen, 2008) (EBICglasso) was used. Pairwise complete observations handled missing data, and the tuning parameter was 0.5.
3. Networks were visualized using qgraph (Epskamp et al., 2012), employing the Fruchterman–Reingold algorithm (Fruchterman & Reingold, 1991), which pulls the most connected nodes to the center of the network. Blue edges indicate positive and red edges indicate negative partial correlations.
4. Network properties included node strength, measuring centrality by summing absolute edge weights connected to each node (Opsahl et al., 2010). Raw scores were displayed (Burger et al., 2023).
5. Stability and accuracy of edge weights and centrality estimates were evaluated via 1,000 bootstrap resamples, generating 95% confidence intervals (CIs) for edge weights (Supplementary Figure S1), testing edge weight differences (Supplementary Figure S2), and comparing centrality estimates (Supplementary Figure S3). Stability was assessed with a case-drop bootstrap, summarized using the correlation stability (CS) coefficient (recommended value >0.5; Supplementary Figure S4; Epskamp, Borsboom, & Fried, 2018).

### Sensitivity analyses

The diagnostic criteria for PGD differ slightly between the DSM-5-TR (American Psychiatric Association, 2022) and ICD-11 (Killikelly & Maercker, 2018), reflecting varying conceptualizations that may influence network analysis results. We conducted sensitivity analyses using PGD criteria from ICD-11, applying the same methods as described above. To facilitate comparison, we used the network layout from the DSM-5 analysis for the ICD-11 network.

## Results

### Sample characteristics

Demographics and loss characteristics of the sample are presented in Table 1.

**Table 1.** Sample Characteristics

Variable	Range	Mean	Std. deviation	Frequency	Percent
Age (in years)	18–85	49.20	12.58		
Months since loss	6–685	56.79	80.86		
Gender					
Male				125	18.0
Female				570	82.0
Relationship of the deceased					
Child				150	21.6
Partner				250	36.0
Sibling				45	6.5
Parent				201	28.9
Close nonrelative				15	2.2
Close other relative				34	4.9
Education level					
No qualifications				28	4.0
High school education				224	32.2
University degree				301	43.3
Postgraduate degree				140	20.1
Mode of death					
Nonviolent				561	80.7
Violent				134	19.3

*Note*: Violent = Initiated by human (in)action.

### *Network estimation*^***1***^

**Figure 1.**
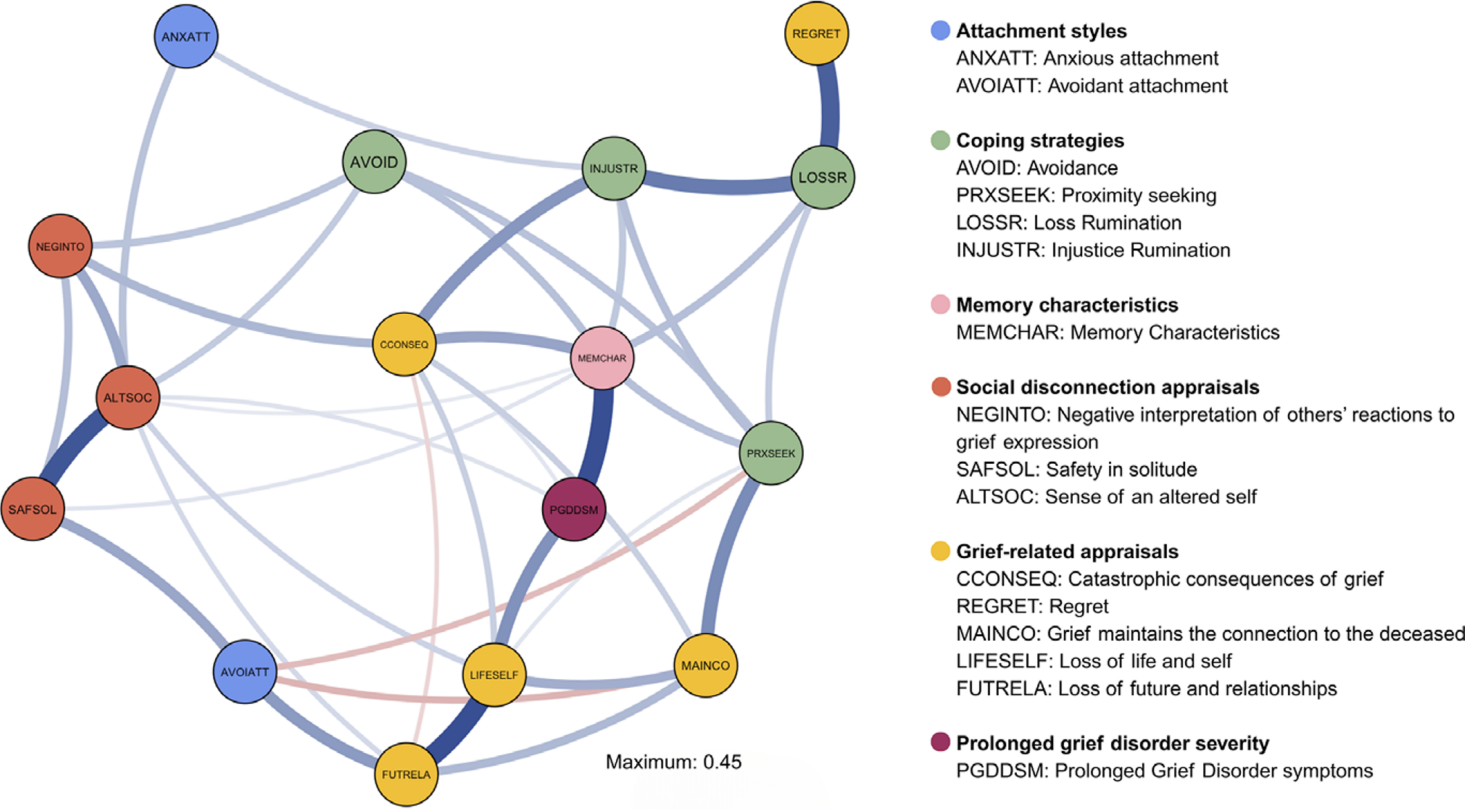
Regularized network structure of the relationship between attachment style, cognitive behavioral components and PGD severity. The red edges indicate negative partial correlations, and the blue edges represent positive partial correlations. Edge thickness indicates the size of the relationship with thicker edge weights indicating a stronger partial correlation. Note: Attachment styles – ANXATT = Anxious attachment; AVOIATT = Avoidant Attachment; Coping Strategies – PROXSEEK = Proximity seeking; LOSSR = Loss Rumination; INJUSTR = Injustice Rumination; AVOID = Avoidance; MEMCHAR = Memory Characteristics; Social Disconnection Appraisals – NEGINTO = Negative interpretation of others reactions to grief expression; ALTSOC = Sense of an altered social self; SAFSOL = Safety in solitude; Grief-Related Appraisals – FUTRELA = Loss of future and relationships; LIFESELF = Loss of life and self, MAINCON = Grief maintains connection to the deceased; CCONSEQ = Catastrophic consequences of grief; REGRET = Regret; PGDDSM = Prolonged Grief Disorder symptoms.

The regularized partial correlation network is shown in Figure 1. Accuracy and stability analyses confirmed reliable interpretation of edge weights and centrality indices (CS-coefficient > 0.5; see Supplementary Materials). Almost all of the edge weights in the network were positive (indicated in blue), with the exception of three pairs (indicated in red).

### Attachment

**Figure 2.**
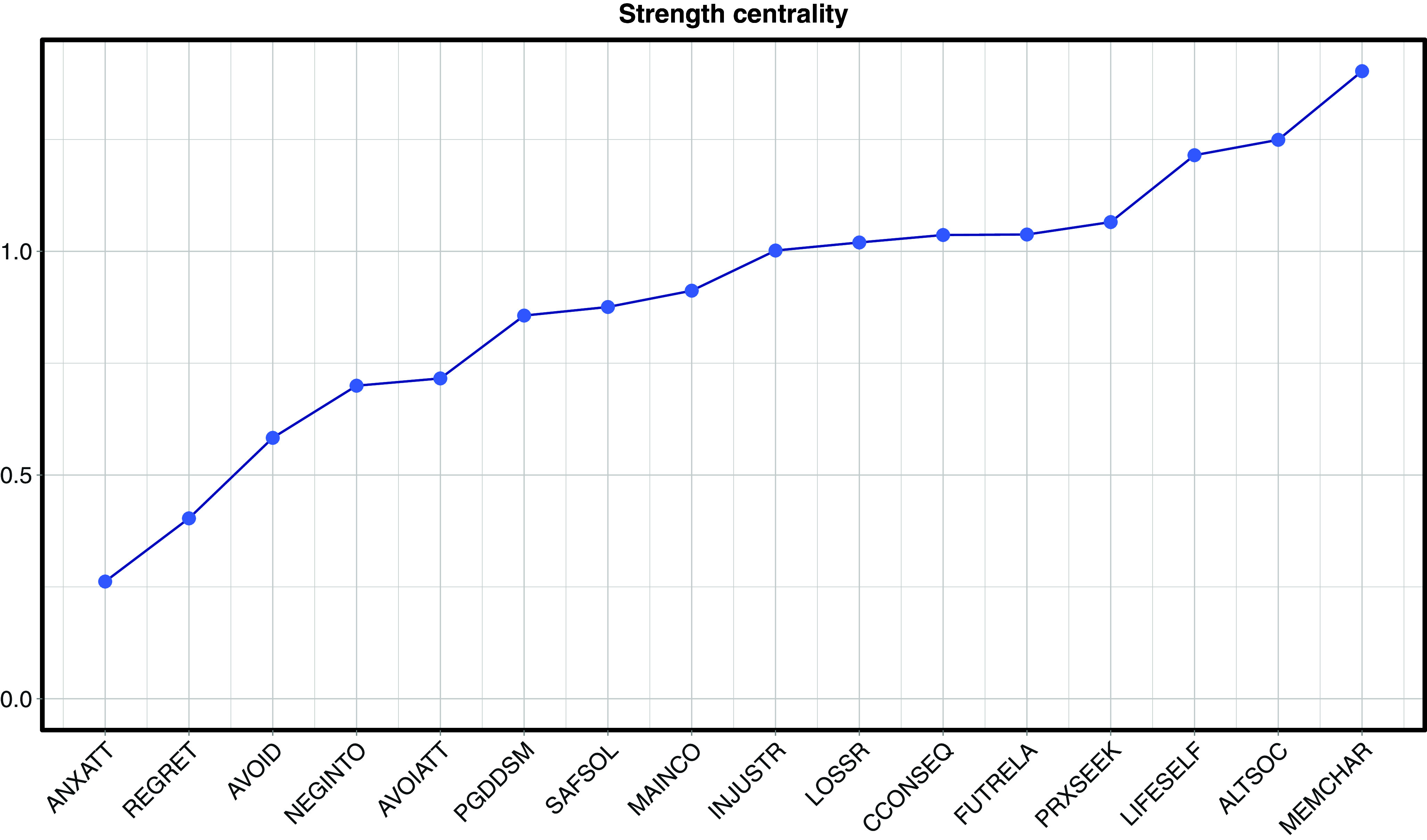
Raw node strength centrality of the 16 variables in the network, from highest (MEMCHAR) to lowest (ANXATT). Note: Attachment styles – ANXATT = Anxious attachment; AVOIATT = Avoidant Attachment; Coping Strategies – PROXSEEK = Proximity seeking; LOSSR = Loss Rumination; INJUSTR = Injustice Rumination; AVOID = Avoidance; MEMCHAR = Memory Characteristics; Social Disconnection Appraisals – NEGINTO = Negative interpretation of others’ reactions to grief expression; ALTSOC = Sense of an altered social self; SAFSOL = Safety in solitude; Grief-Related Appraisals – FUTRELA = Loss of future and relationships; LIFESELF = Loss of life and self, MAINCON = Grief maintains connection to the deceased; CCONSEQ = Catastrophic consequences of grief; REGRET = Regret; PGDDSM = Prolonged Grief Disorder symptoms.

The two attachment nodes (anxious and avoidant) appeared on the periphery of the network and demonstrated no direct relationship with the PGD node. Furthermore, they did not share a direct edge with each other or with any of the same remaining nodes, indicating that they likely have a differential influence on the PGD network. Both had low centrality (Figure 2 and Supplementary Figure S4). Anxious attachment positively correlated with injustice rumination (0.11) and altered social self (0.15), indicating greater anxious attachment relates to social disruption and increased rumination about the unfairness of loss. Avoidant attachment positively correlated with loss of future and relationships (0.25) and safety in solitude (0.22), indicating avoidant attachment associates with a preference for solitude and perceived future and relational loss. Negative associations with proximity seeking (−0.12) and grief maintains connection to the deceased appraisals (−0.13) suggest that greater avoidant attachment relates to less fear of losing connection to the deceased and lower engagement in closeness-seeking behaviors.

### Prolonged grief disorder

PGD had four direct positive edges; the strongest was with memory characteristics (0.45), significantly stronger than all but three (the next three strongest) edges (Supplementary Figure S2). PGD also positively linked with three appraisals nodes: loss of future and relationships (0.27), catastrophic grief consequences (0.06), and an altered social self (0.07).

### Highly central nodes

Memory characteristics were the most central node (eight connections, highest strength; Figure 2), significantly more central than all but altered social self and loss of life and self-appraisal nodes (Supplementary Figure S3). This highlights the influence of memory processes on PGD severity and a multitude of negative consequences after a bereavement. Memory characteristics connected to all four maladaptive coping strategies: avoidance (0.15), proximity seeking (0.16), loss rumination (0.16), and injustice rumination (0.14).

Loss of life and self-appraisals, also highly central, directly connected to PGD and four other appraisal nodes: loss of future and relationships (0.45), grief maintains connection to the deceased (0.20), catastrophic of consequences grief (0.12), altered social self (0.11), plus one coping strategy (proximity seeking, 0.06).

Altered social self-appraisals, directly connected to PGD (0.07), were the second most central node, connecting to six others, including memory characteristics, and loss of life and self-appraisals. It also linked to loss of future and relationships appraisals (0.05), avoidant coping (0.13), negative interpretation of others’ reactions to grief expression (0.21), and safety in solitude (0.43). This suggests that the sense of an altered social self may mediate or amplify other variables’ impact on PGD symptoms.

### Connections between mechanisms

Following memory characteristics and PGD, the next strongest associations were between related appraisal nodes. Loss of life and self strongly connected to loss of relationships and future (0.45; Grief Appraisals Scale). Altered social self strongly linked to safety in solitude (0.43; Social Disconnection Scale). Loss rumination had strong associations with regret (0.40) and injustice rumination (0.33), both linking to memory characteristics – the strongest node connected to PGD. Social disconnection appraisals (altered social self, negative interpretations of other’s reactions to grief expression) were directly linked to avoidant coping (0.13 and 0.15). Appraisals about grief maintaining connection to the deceased correlated with proximity seeking behaviors (0.29), while appraisals about the catastrophic consequences of grief appraisals correlated with injustice rumination (0.26).

### Sensitivity analyses

Sensitivity analyses using ICD-11 PGD criteria (Supplementary Materials) showed highly similar networks (75.83% identical values, r = 0.97 correlation between partial correlation matrices). However, three DSM-5-TR network edges were absent in the ICD-11 network: safety in solitude and altered social self did not link to memory characteristics and PGD, respectively, and anxious attachment did not link to injustice rumination.

## Discussion

The present study sought to examine how anxious and avoidant attachment styles relate to PGD symptom severity, as defined by DSM-5-TR criteria, when considered within a network of cognitive-behavioral grief-related mechanisms. Neither anxious nor avoidant attachment was found to have a direct relationship with PGD symptoms; however, our findings reveal that each attachment style had associations to distinct cognitive-behavioral processes, and in turn prolonged grief. This supports the hypothesis that attachment styles may indirectly shape PGD symptoms by modulating key social and cognitive mechanisms rather than directly driving grief outcomes.

### Attachment styles and indirect influences on PGD

#### Anxious attachment

Anxious attachment was positively associated with beliefs about being socially altered following loss and with injustice-related rumination. These connections align with the view that anxious attachment heightens emotional distress and amplifies cognitive intrusions (Yousefi & Ashouri, 2023). Moreover, the strong association with disruptions in social functioning beliefs suggests they may mediate the relationship between anxious attachment and PGD symptoms (Janshen et al., 2024). Thus, while individuals with anxious attachment may remain emotionally engaged with their grief, their heightened sensitivity to perceived injustices and altered social identity could intensify the distressing aspects of bereavement.

#### Avoidant attachment

Avoidant attachment was negatively associated with both appraisals that grief maintains connection to the deceased and proximity-seeking behaviors, suggesting that avoidantly attached individuals may suppress grief-related thoughts and behaviors that foster closeness to the deceased. These results align with previous findings that avoidant attachment is linked to both depressive avoidance (e.g. withdrawing from potentially adaptive activities) and anxious avoidance (e.g. avoiding reminders of the loss) (Boelen & van den Bout, 2010). Although this pattern could indirectly reduce some aspects of PGD – particularly those tied to seeking active closeness to the deceased – our network also revealed that avoidant attachment is positively linked with beliefs about social disconnectedness (i.e. feeling only safe when alone) and beliefs about a loss of relationships and a bright future. These nodes, in turn, were strongly connected to the altered social self and loss of life and self-appraisals, which are directly connected with PGD. This pattern is consistent with studies suggesting that avoidant attachment fosters emotional disengagement and a disruption in the sense of self, thereby perpetuating a sense of hopelessness and potentially contributing to chronic grief (Maccallum & Bryant, 2013).

This duality may help explain the mixed findings in the literature. Some studies have reported negative associations between avoidant attachment and grief outcomes (Delespaux, Ryckebosch-Dayez, Heeren, & Zech, 2013; LeRoy et al., 2020; Smigelsky, Bottomley, Relyea, & Neimeyer, 2020), whereas others have found positive associations (Boelen & Klugkist, 2011; Boelen & van den Bout, 2010; Field & Filanosky, 2009; Gegieckaite & Kazlauskas, 2022). These inconsistencies may stem from differences in how avoidant attachment and grief are measured or conceptualized, as well as variations in sample characteristics (e.g. relationship to the deceased, cultural context). It is also possible that studies focusing on the ‘avoidance of distress’ aspect capture a different facet of avoidant attachment than those emphasizing ‘social losses and disconnection’, thereby influencing whether significant associations, and in which direction, are observed.

A number of studies have reported that psychological interventions (e.g. Cognitive-Behavioral Therapy [CBT]) can lead to modest reductions in both anxious and avoidant attachment (Levy et al., 2006; Strauß et al., 2018); these effects are typically small to moderate. However, recent research demonstrated enhanced effects of CBT for PGD symptoms in individuals who have high attachment anxiety (Schmidt, Treml, Linde, Peterhänsel, & Kersting, 2022). In line with these findings, our study raises the possibility that targeting the specific mechanisms related to attachment style, such as emotional disengagement in avoidant individuals or social disconnection in those with anxious attachment, may show promise in enhancing the efficacy of interventions for PGD and should be investigated in future research.

### Memory characteristics

A key finding of our analysis was the prominent role of memory characteristics. With the strongest direct connection to PGD symptoms (edge weight of 0.45) and a high connectivity with eight other nodes, loss memory characteristics were shown to act as a central component within the network. This observation is consistent with previous longitudinal research from a separate dataset that has highlighted the centrality of maladaptive memory processes in sustaining severe grief (Smith & Ehlers, 2020; Smith et al., 2022), and psychological models of PGD (Boelen, van den Hout, & van den Bout, 2006; Maccallum & Bryant, 2013; Shear et al., 2007) and PTSD (Ehlers & Clark, 2000) that propose that the manner in which individuals recall and process their loss increases the likelihood of developing a variety of adverse mental health outcomes following bereavement.

The high centrality of memory characteristics raises the possibility that these processes may not only directly relate to the intensity of PGD symptoms but may also indirectly relate to other nodes in the network, including coping strategies and cognitive appraisals. In our network, memory characteristics were connected to all four unhelpful coping strategies assessed, indicating an influential role of memory processes in relation to the coping mechanisms employed. For example, memory distortions and persistent intrusive recollections may reinforce negative appraisals about the catastrophic consequences of loss, thereby fueling rumination about counterfactuals, the unfairness of the loss, and its consequences. Theoretically, this could exacerbate the severity of PGD symptoms and hinder adaptive coping. This result is supported by longitudinal findings demonstrating that memory characteristics in the first 6 months of loss significantly predict the magnitude of maladaptive coping 6 months later (Smith & Ehlers, 2021a). However, it is also possible that the effects may operate in the opposite direction. For example, repetitive thinking about the loss and proximity seeking behaviors may serve to trigger painful loss memories. More longitudinal studies are needed to test the directionality of the observed effects.

Taken together with previous longitudinal findings, these results suggest that interventions targeting loss memory integration – for example, through memory updating, narrative reconstruction or imaginal reliving of the death narrative are likely to be the most influential in reducing PGD symptoms (Bryant et al., 2014; Duffy & Wild, 2023; Reitsma, Boelen, de Keijser, & Lenferink, 2023; Shear et al., 2016).

#### Social disconnection

Both attachment styles were linked to social disconnection nodes highlighting the importance of social processes in grief. Alterations to an individual’s social self were highly central to the network. It was the only social disconnection node with connections to all other categories of cognitive behavioral nodes (i.e. memory characteristics, coping strategies, and four other negative appraisals nodes – loss of life and self, relationships and future, a sense there is safety in solitude and negative interpretations of others’ reactions to grief expression). It also had a small direct connection with PGD (0.07) as defined in DSM-5, but not ICD-11 (see the Appendix). This supports previous research that found social disconnection to be a key mechanism in the development and maintenance of psychological distress after loss (Smith, Wild, & Ehlers, 2020b), predictive of a reduction in social contact after loss (Wanza et al., 2023) and the strongest predictor of PGD in a sample of individuals who lost loved ones to COVID-19 in the ICU during the pandemic (Rodriguez-Villar et al., 2024). These findings also map on to a recent micro-sociological perspective that acknowledges the social deprivations created by a bereavement play a significant role for these reduction in grief adaptation (Maciejewski, Falzarano, She, Lichtenthal, & Prigerson, 2021).

Given that both anxious and avoidant attachment styles may contribute to social withdrawal—albeit through different pathways—future research should explore interventions aimed at reducing social isolation, enhancing social support, and promoting adaptive self-views, as these may have downstream benefits for individuals at risk of or suffering from PGD. Incorporating family or community resources, as well as psychoeducation that addresses stigmatizing beliefs about grief (Eisma, 2018), may help break the cycle of social disconnection.

Despite these insights, several limitations must be acknowledged. First, this study is cross-sectional, which restricts conclusions about causality or the temporal progression of variables. No definitive statements about causality or the directionality of effects can be made from our findings and future research using longitudinal data should aim to clarify the direction of these relationships. Secondly, the sample was predominantly White and female, limiting the generalizability of these findings to other cultural contexts. However, a recent systematic review and meta-analysis found that being female was a significant risk factor for PGD meaning women are more likely to be affected by the disorder (Buur et al., 2024) making the discrepancy in gender profile less surprising. The reliance on self-report measures introduces the potential reporting biases and precludes any conclusions about diagnostic levels of PGD. Our attachment measure, although very commonly used in the field, had an acceptable internal consistency. Finally, this study focused on DSM-5-TR criteria in the main body of the paper, and there are small differences in how the network manifests using the ICD-11 PGD criteria. However, our results showed an extremely high similarity between the two conceptualizations networks. Clinically, our findings underscore the potential benefits of tailoring interventions to target specific cognitive and social processes associated with PGD. For example, it would be valuable for future research to investigate whether treatments aimed at integrating maladaptive loss memories or enhancing social reconnection are especially effective for individuals with pronounced avoidant attachment, while interventions addressing rumination and distorted social perceptions may prove more beneficial for those with high attachment anxiety. Future studies employing longitudinal designs and more diverse samples, as well as multimethod approaches, are needed to further elucidate these complex pathways and refine treatment strategies. For example, measuring attachment and related mechanisms over multiple time points, or assessing changes pre- and postintervention, could provide valuable insights into the dynamic nature of these processes.

In summary, while attachment styles may not directly drive prolonged grief, their indirect influence through differential links to key cognitive-behavioral mechanisms is evident. By identifying the centrality of memory characteristics and the role of social disconnection in sustaining PGD, this study contributes to a more nuanced understanding of grief and offers promising avenues for targeted therapeutic intervention.

## Supporting information

Smith et al. supplementary material Smith et al. supplementary material

## Footnotes

1 See supplementary materials for the network estimation of the ICD-11 PGD diagnosis, which did not substantially differ from the DSM diagnoses.

## Supplementary material

To view supplementary material for this article, please visit http://doi.org/10.1017/S0033291725101669.
