# Supplementary material for: Unraveling attachment – A network analysis of the cognitive pathways linking attachment and prolonged grief
Source: Psychol Med. 2025 Sep 19;55:e276. doi: 10.1017/S0033291725101669 (PMC13054909; doi:10.1017/S0033291725101669)
Supplement: Smith et al. supplementary material [file S0033291725101669sup001.pdf]

Supplementary Materials

Table S1: Specific edge weights (DSM network)……………………..………..............……..2

Table S2: Specific edge weights (ICD network)……………………..………………..……….3

Figure S1: ICD-11 Network……………………………………………………………………4

Stability Analysis (DSM network)……………..……………………...…………………...…...…5

Figure S2 (Edge Weight Stability)……...………………………………………………5

Figure S3 (Edge Weight Difference Test)…………………………………….…………..6

Figure S4 (Centrality Difference Test)…………………………………………………7

Figure S5 (Centrality Stability)……….....………………………………………………8

Stability Analysis (ICD network)…………..…………………….....……………..……..……9

Figure S6 (Edge Weight Stability)……...………………………………………………9

Figure S7 (Edge Weight Difference Test)…………………..……………………………10

Figure S8 (Centrality Difference Test)……………………..……………………….....11

Figure S9 (Centrality Stability)……….....…………………………………………..……12

Table S1

Specific Edge Wights for Network with DSM Prolonged Grief Conceptualisation

	1	2	3	4	5	6	7	8	9	10	11	12	13	14	15	16
1. CCONSEQ																
2. REGRET	0.00															
3. MAINCO	0.12	0.00														
4. LIFESELF	0.12	0.00	0.20													
5. FUTRELA	-0.07	0.00	0.17	0.45												
6. AVOID	0.00	0.00	0.00	0.00	0.00											
7. PRXSEEK	0.00	0.00	0.29	0.06	0.00	0.15										
8. LOSSR	0.00	0.40	0.00	0.00	0.00	0.00	0.13									
9. INJUSTR	0.26	0.00	0.00	0.00	0.00	0.00	0.16	0.33								
10. MEMCHAR	0.23	0.00	0.00	0.00	0.00	0.15	0.16	0.16	0.14							
11. NEGINTO	0.18	0.00	0.00	0.00	0.00	0.15	0.00	0.00	0.00	0.00						
12. SAFSOL	0.00	0.00	0.00	0.00	0.00	0.00	0.00	0.00	0.00	0.07	0.16					
13. ALTSOC	0.00	0.00	0.00	0.11	0.09	0.13	0.00	0.00	0.00	0.05	0.21	0.43				
14. ANXATT	0.00	0.00	0.00	0.00	0.00	0.00	0.00	0.00	0.11	0.00	0.00	0.00	0.15			
15. AVOIATT	0.00	0.00	-0.13	0.00	0.25	0.00	-0.12	0.00	0.00	0.00	0.00	0.22	0.00	0.00		
16. PGDSM	0.06	0.00	0.00	0.27	0.00	0.00	0.00	0.00	0.00	0.45	0.00	0.00	0.07	0.00	0.00	

Note: 1. CCONSEQ = Catastrophic consequences of grief; 2. REGRET; 3. MAINCON = Grief maintains the connection to the deceased; 4 LIFESELF = Loss of life and self; 5. FUTRELA = Loss of future and relationships;; 6 AVOID = Avoidance; 7. PROXSEEK = Proximity seeking; 8. LOSSR = Loss Rumination; ; 9. INJUSTR = Injustice Rumination; 10. MEMCHAR = Memory Characteristics; 11. NEGINTO = Negative interpretation of others' reactions to grief expression; 12. SAFSOL = Safety in solitude. 13. ALTSOC = Sense of an altered social self; 14. ANXATT = Anxious attachment; 15. AVOIATT = Avoidant Attachment;16. PGDDSM = Prolonged Grief Disorder symptoms (DSM-5). Green colour represents positive partial correlations, while the orange values are negative partial correlations.

Table S2

Specific Edge Wights for Network with ICD Prolonged Grief Conceptualisation

	1	2	3	4	5	6	7	8	9	10	11	12	13	14	15	16
1. CCONSEQ																
2. REGRET	0.00															
3. MAINCO	0.11	0.00														
4. LIFESELF	0.12	0.00	0.20													
5. FUTRELA	0.00	0.00	0.16	0.46												
6. AVOID	0.00	0.00	0.00	0.00	0.00											
7. PRXSEEK	0.00	0.00	0.29	0.07	0.00	0.15										
8. LOSSR	0.00	0.39	0.00	0.00	0.00	0.00	0.12									
9. INJUSTR	0.25	0.00	0.00	0.00	0.00	0.00	0.16	0.32								
10. MEMCHAR	0.21	0.00	0.00	0.07	0.00	0.16	0.16	0.10	0.12							
11. NEGINTO	0.18	0.00	0.00	0.00	0.00	0.15	0.00	0.00	0.00	0.00						
12. SAFSOL	0.00	0.00	0.00	0.00	0.00	0.00	0.00	0.00	0.00	0.00	0.16					
13. ALTSOC	0.00	0.00	0.00	0.12	0.09	0.13	0.00	0.00	0.00	0.06	0.21	0.42				
14. ANXATT	0.00	0.00	0.00	0.00	0.00	0.00	0.00	0.00	0.11	0.00	0.00	0.00	0.14			
15. AVOIATT	0.00	0.00	-0.12	0.00	0.24	0.00	-0.12	0.00	0.00	0.00	0.00	0.21	0.00	0.00		
16. PGICD	0.09	0.00	0.00	0.15	0.07	0.00	0.00	0.11	0.00	0.44	0.00	0.00	0.00	0.00	0.00	

Note: 1. CCONSEQ = Catastrophic consequences of grief; 2. REGRET; 3. MAINCON = Grief maintains the connection to the deceased; 4 LIFESELF = Loss of life and self; 5. FUTRELA = Loss of future and relationships;; 6 AVOID = Avoidance; 7. PROXSEEK = Proximity seeking; 8. LOSSR = Loss Rumination; ; 9. INJUSTR = Injustice Rumination; 10. MEMCHAR = Memory Characteristics; 11. NEGINTO = Negative interpretation of others' reactions to grief expression; 12. SAFSOL = Safety in solitude. 13. ALTSOC = Sense of an altered social self; 14. ANXATT = Anxious attachment; 15. AVOIATT = Avoidant Attachment;16. PGDICD= Prolonged Grief Disorder symptoms (ICD-11). Green colour represents positive partial correlations, while the orange values are negative partial correlations.

Figure S1

The Regularized Network Structure for PGD ICD-11

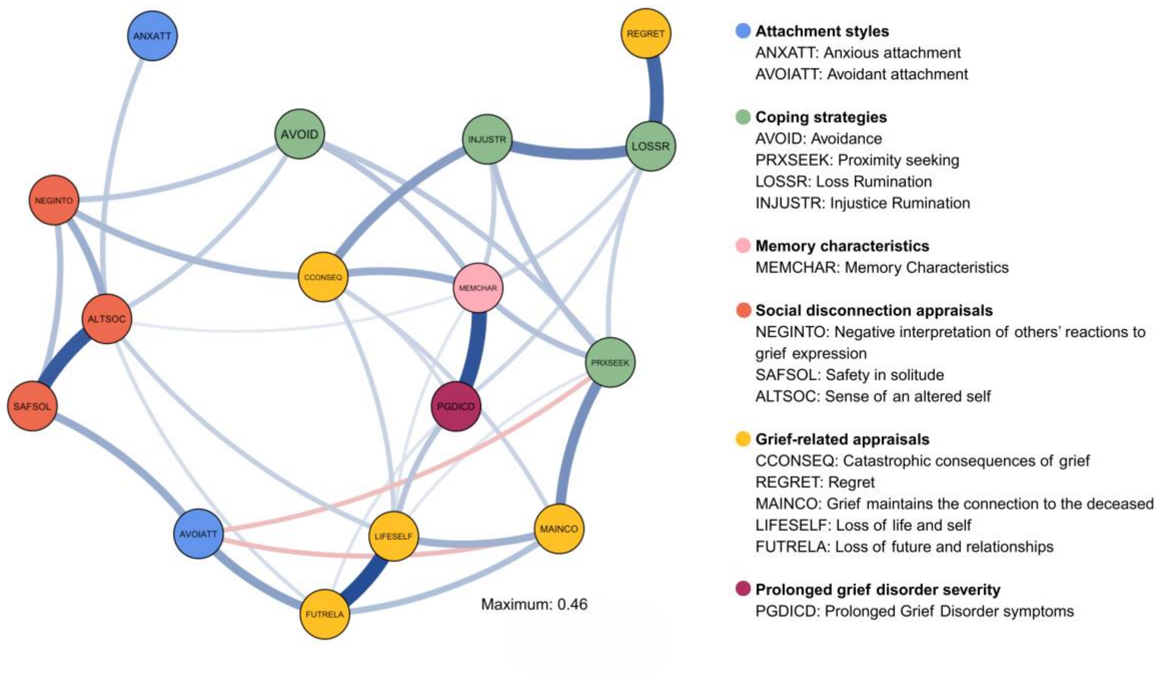

The red edges indicate negative partial correlations, and the blue edges represent positive partial correlations. Edge thickness indicates the size of the relationship with thicker edge weights indicating a stronger partial correlation. PGDICD= Prolonged Grief Disorder symptoms; ANXATT = Anxious attachment; AVOIATT = Avoidant Attachment; MEMCHAR = Memory Characteristics; Coping Strategies - PROXSEEK = Proximity seeking; LOSSR = Loss Rumination; INJUSTR = Injustice Rumination; AVOID = Avoidance; Grief-Related Appraisals – FUTRELA = Loss of future and relationships; LIFESELF = Loss of life and self, MAINCON = Grief maintains the connection to the deceased; CCONSEQ = Catastrophic consequences of grief; REGRET = Regret; Social Disconnection – NEGINTO = Negative interpretation of others' reactions to grief expression; ALTSOC = Sense of an altered social self; SAFSOL = Safety in solitude.

Figure S2

Stability of Edge Weights Based on 1000 Bootstraps (DSM)

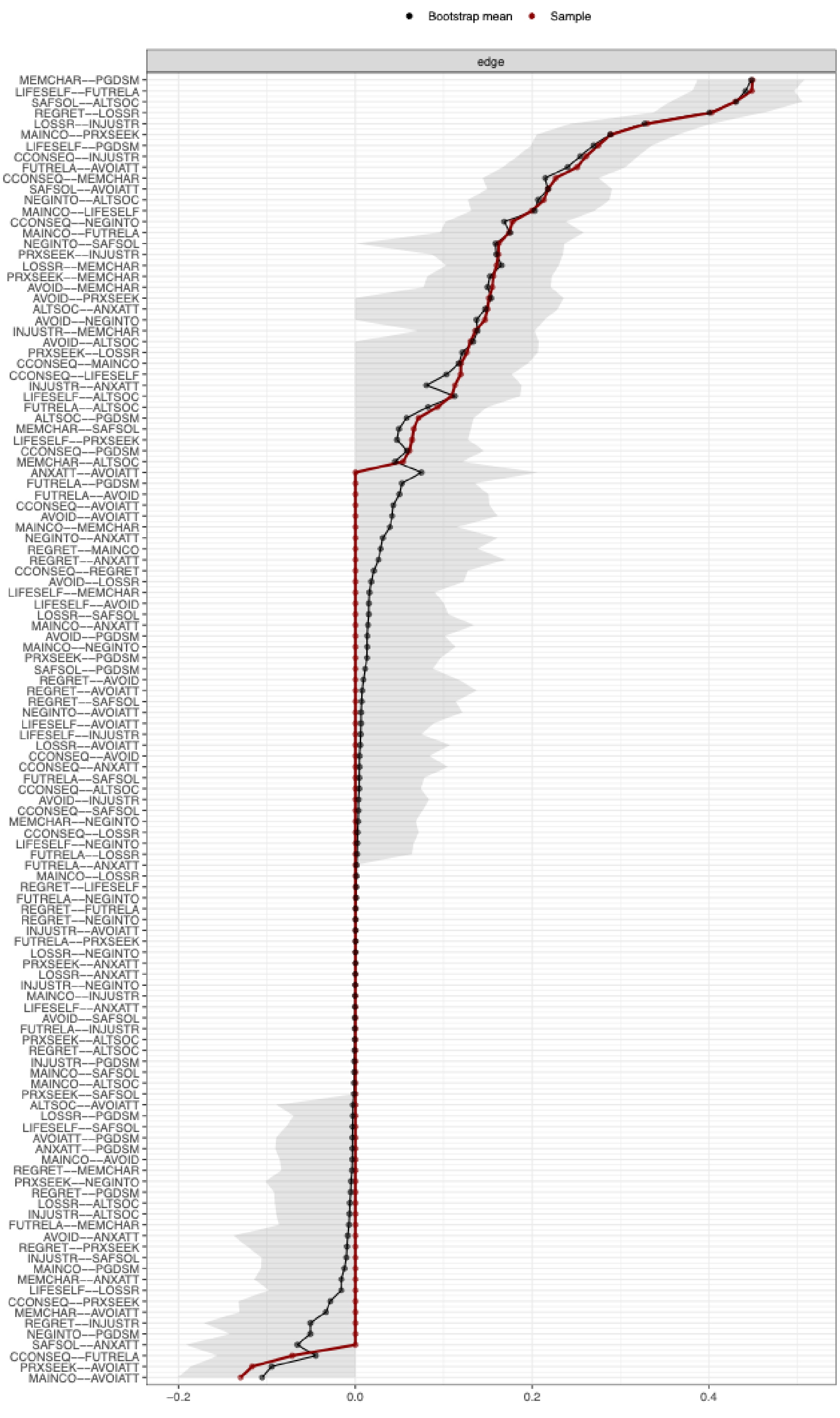

Figure S3

Edge Weight Difference Test based on 1000 Bootstraps (DSM)

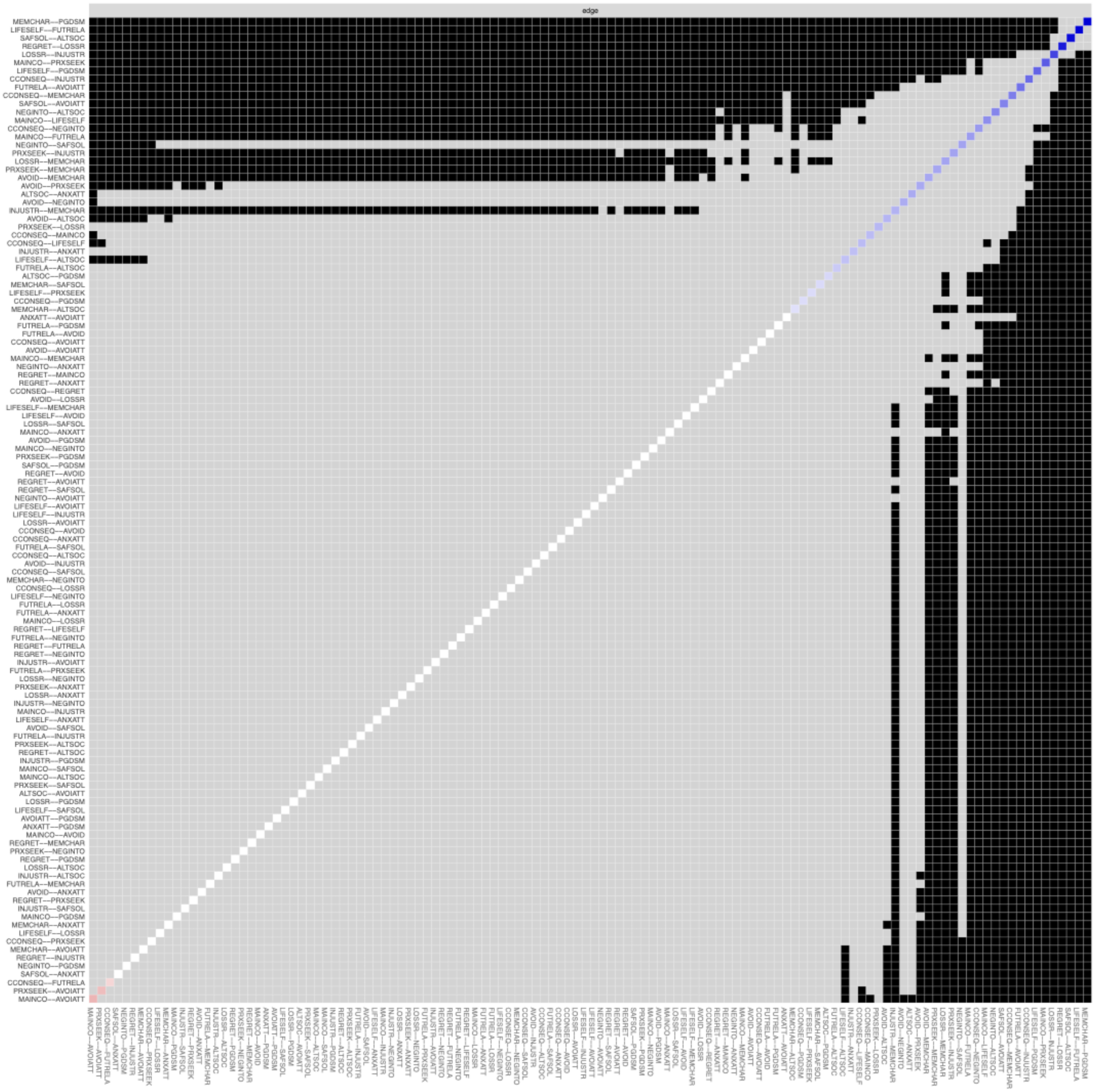

Note. Each row represents an edge in the network. The coloured squares (black) represent a significant pairwise test (whether one edge is significantly different from another), whereas the grey coloured squares are non-significant differences ($\alpha = 0.05$).

Figure S4

Strength Centrality Difference Test based on 1000 Bootstraps (DSM)

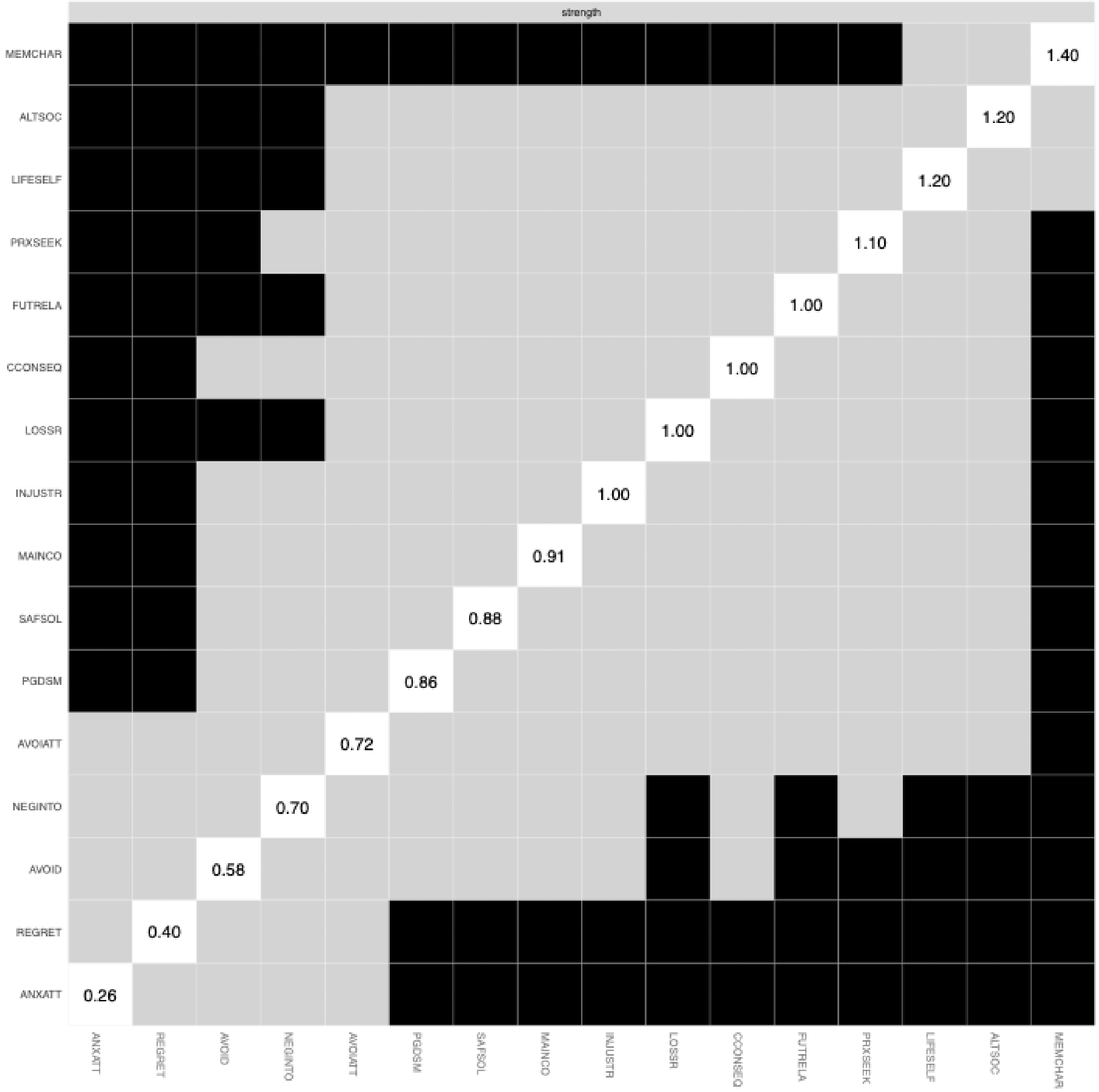

Note. The centrality metric for each variable is presented at the diagonal. Black squares represent significant differences ($\alpha = 0.05$) between two node's centrality values, whereas grey squares are non-significant differences.

Figure S5

Stability of the Strength Centrality Estimates based on 1000 non-parametric Bootstraps (DSM)

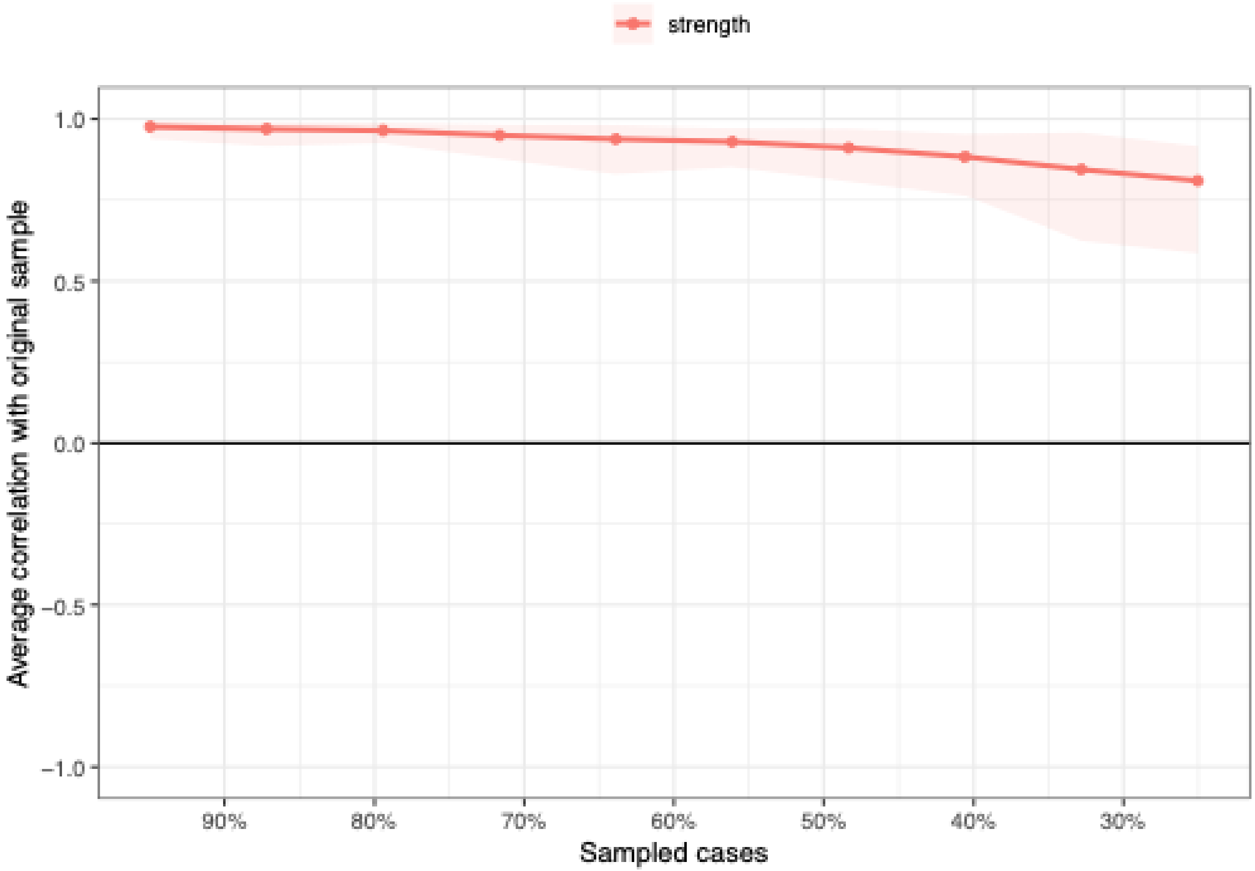

Note. This figure shows the stability of the centrality indices across subsets of the sample. The case-drop bootstrap procedure consecutively drops more and more participants (the x-axis represents percentage (%) of participants remaining), each time calculating centrality metrics and correlates them with that of the original sample. Preferably, the correlation stability coefficient (CS), marked by the red line, should be above 0.5.

Figure S6

Stability of Edge Weights Based on 1000 Bootstraps (ICD)

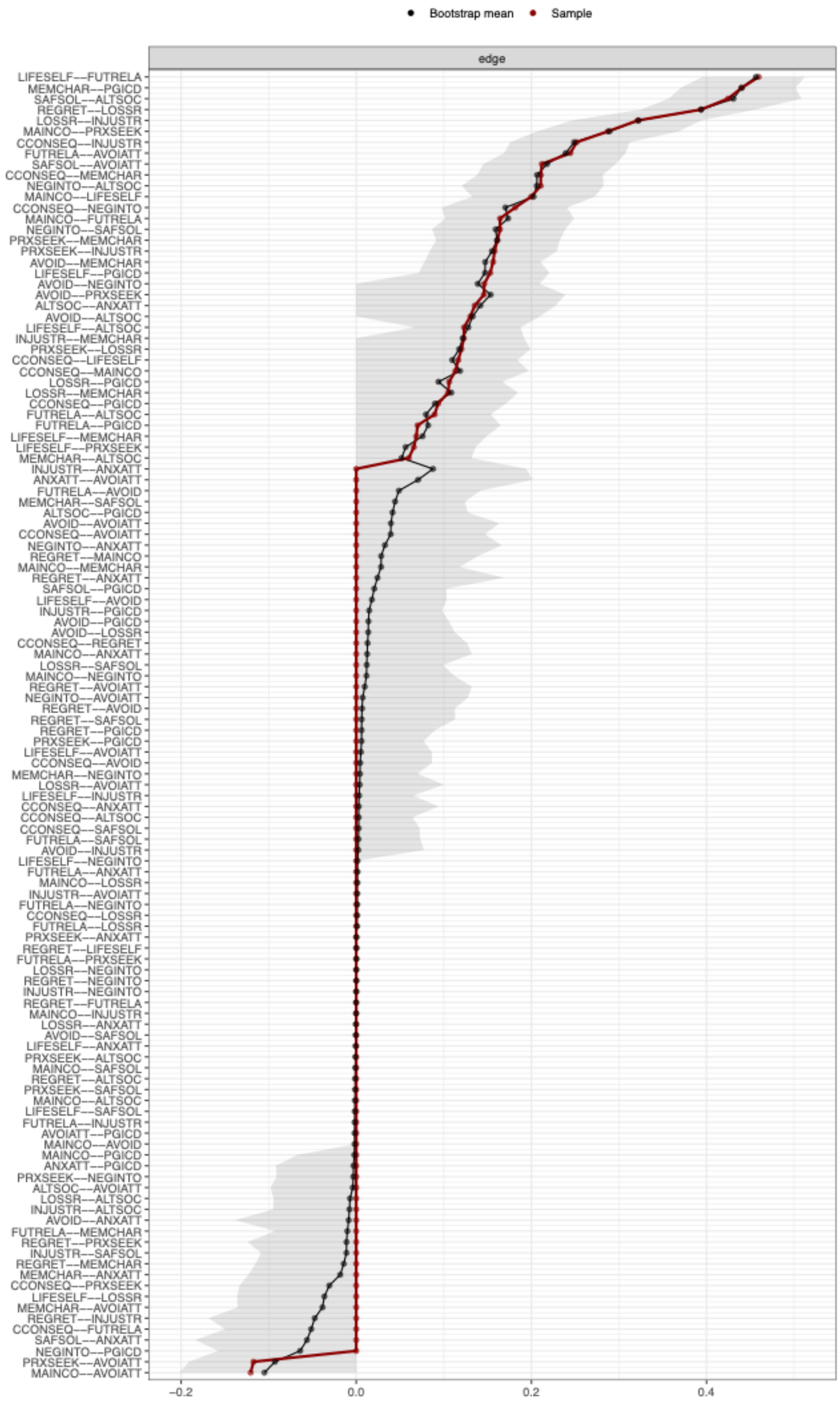

Figure S7

Edge Weight Difference Test based on 1000 Bootstraps (ICD)

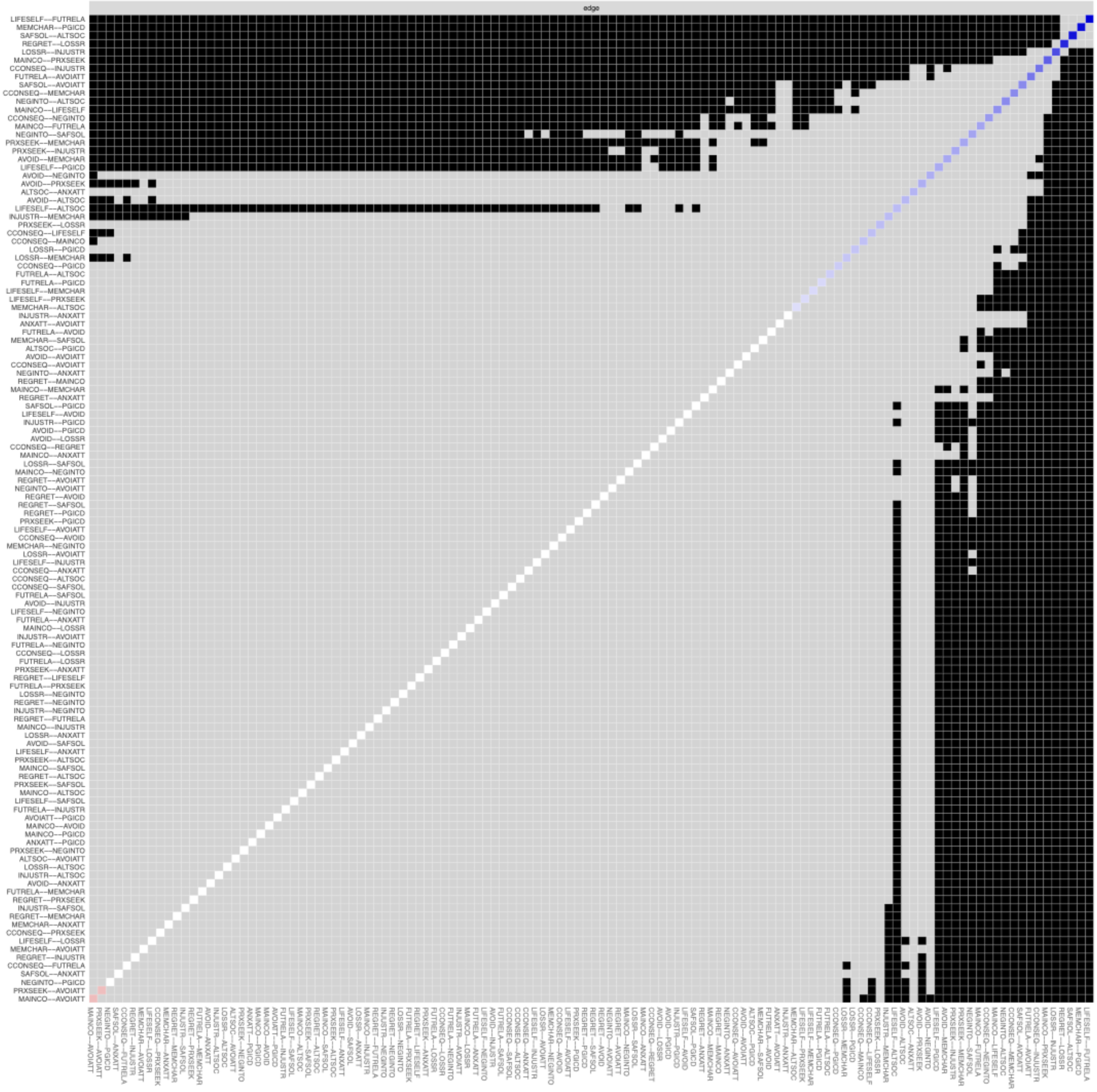

Note. Each row represents an edge in the network. The coloured squares (black) represent a significant pairwise test (whether one edge is significantly different from another), whereas the grey coloured squares are non-significant differences ($\alpha = 0.05$).

Figure S8

Strength Centrality Difference Test based on 1000 Bootstraps (ICD)

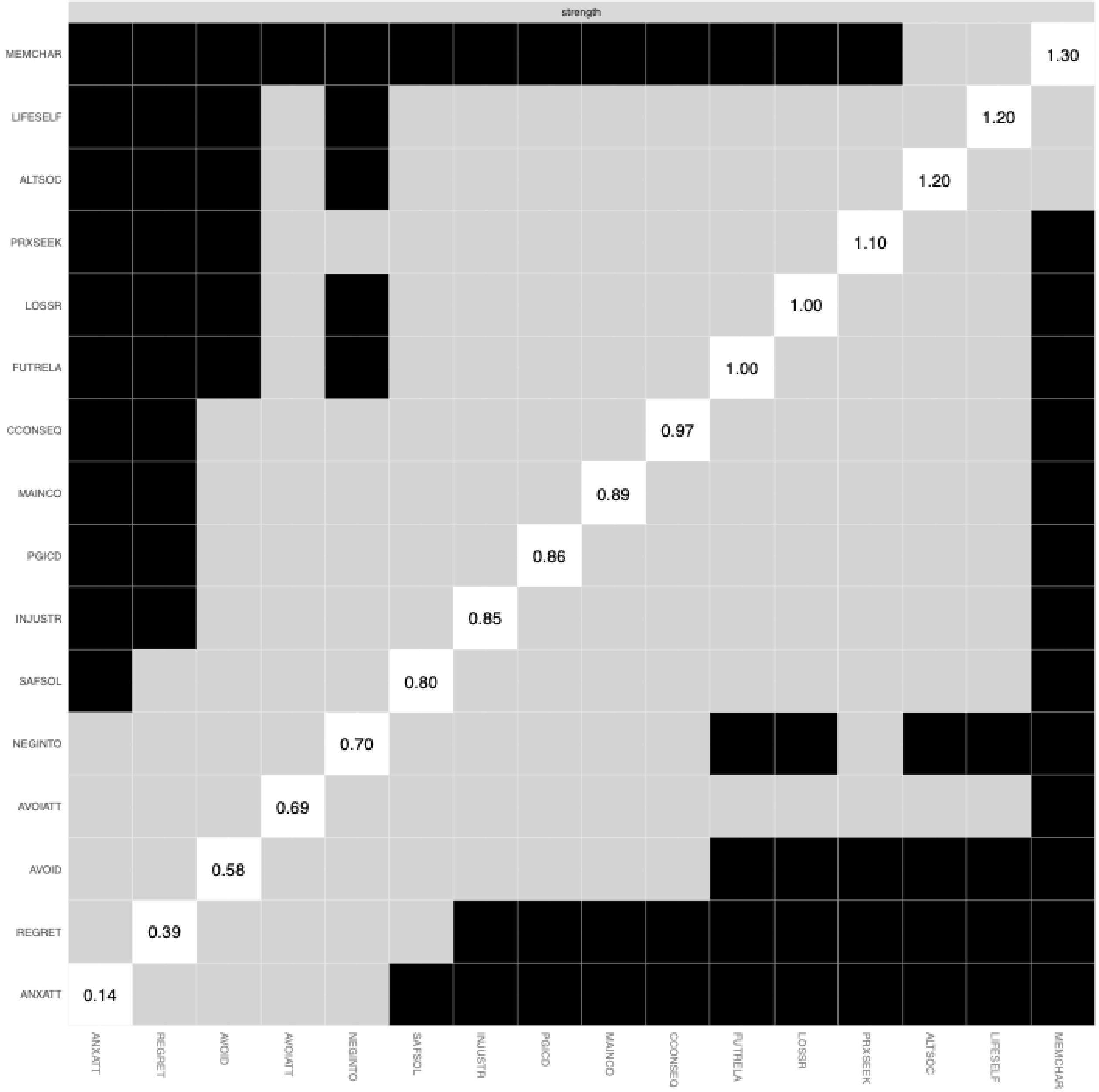

Note. The centrality metric for each variable is presented at the diagonal. Black squares represent significant differences ($\alpha = 0.05$) between two node's centrality values, whereas grey squares are non-significant differences.

Figure S9

Stability of the Strength Centrality Estimates based on 1000 non-parametric Bootstraps (ICD)

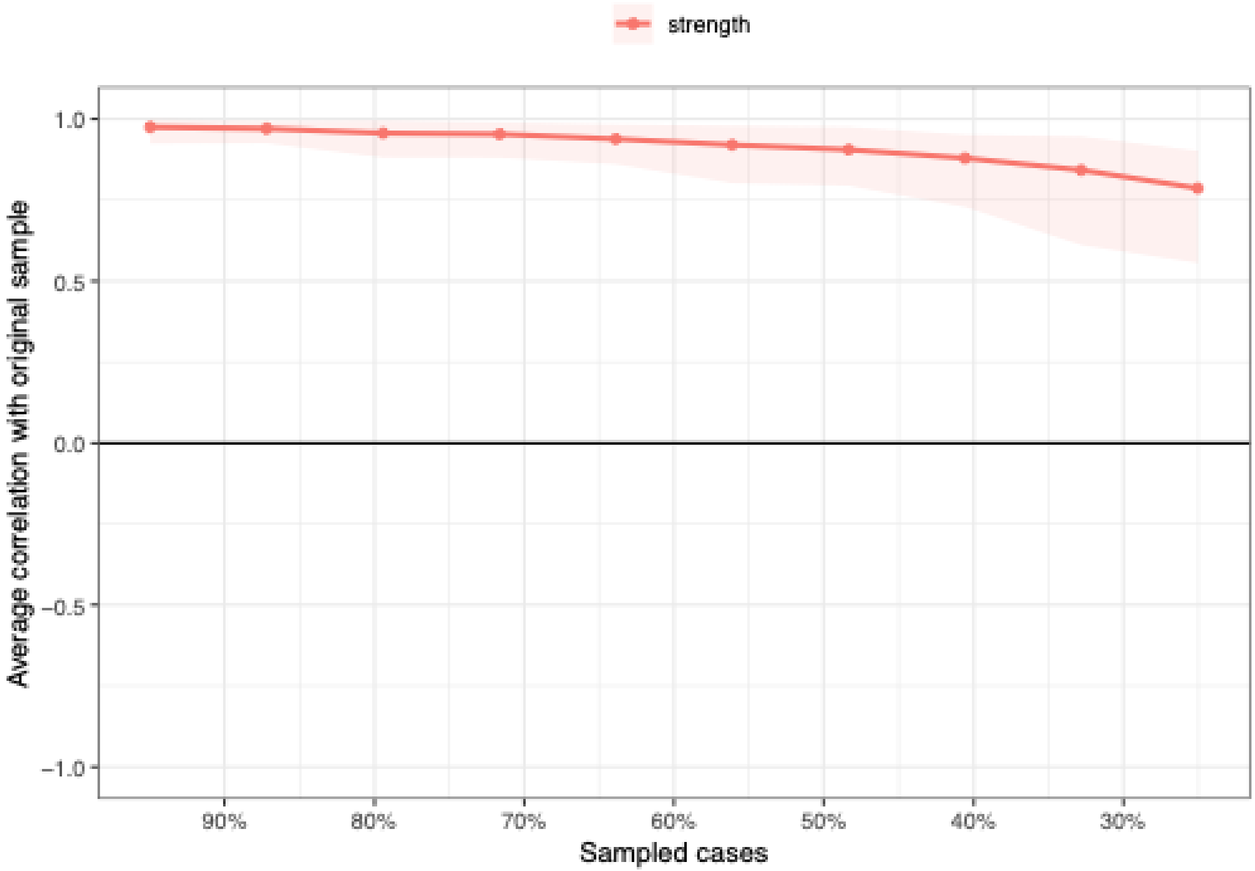

Note. This figure shows the stability of the centrality indices across subsets of the sample. The case-drop bootstrap procedure consecutively drops more and more participants (the x-axis represents percentage (%) of participants remaining), each time calculating centrality metrics and correlates them with that of the original sample. Preferably, the correlation stability coefficient (CS), marked by the red line, should be above 0.5.
